# The Impact of Chronic Comorbidities on Outcomes in Acute Exacerbations of Idiopathic Pulmonary Fibrosis

**DOI:** 10.3390/life14010156

**Published:** 2024-01-21

**Authors:** Saqib H. Baig, Erika J. Yoo

**Affiliations:** Division of Pulmonary, Allergy and Critical Care Medicine, Jane and Leonard Korman Respiratory Institute, Sidney Kimmel Medical College, Thomas Jefferson University, 211 South 9th Street, Suite 401, Philadelphia, PA 19107, USA; erika.yoo@jefferson.edu

**Keywords:** idiopathic pulmonary fibrosis, mortality, length of stay

## Abstract

Introduction: Idiopathic pulmonary fibrosis is a chronic progressive lung disease of unknown cause with a high associated mortality. We aimed to compare the impact of chronic medical conditions on hospital outcomes of patients with acute exacerbations of idiopathic pulmonary fibrosis (AE-IPF). Methods: This was a retrospective cohort study using the NIS database from 2016 to 2018. We included patients aged 60 and older hospitalized in academic medical centers with the diagnoses of IPF and acute respiratory failure. We examined factors associated with hospital mortality and length of stay (LOS) using survey-weighted multivariate logistic and negative binomial regression. Results: Out of 4975 patients with AE-IPF, 665 (13.4%) did not survive hospitalization. There was no difference in the mean age between survivors and non-survivors. Patients were more likely to be male, predominantly white, and have Medicare coverage. Most non-survivors were from households with higher median income. Hospital LOS was longer among non-survivors than survivors (9.4 days vs. 9.8 days; *p* < 0.001). After multivariate-logistic regression, diabetes was found to be protective (aOR 0.62, 95% CI 0.50–0.77; *p* < 0.0001) while chronic kidney disease (CKD) conferred a significantly higher risk of death after AE-IPF (aOR 6.85, 95% CI 1.90–24.7; *p* = 0.00). Our multivariate adjusted negative binomial regression model for LOS identified obesity (IRR 0.85, 95% CI 0.76–0.94; *p* ≤ 0.00) and hypothyroidism (IRR 0.90, 95% CI 0.83–0.98; *p* = 0.02) to be associated with shorter hospital LOS. Conclusions: Our results suggest that CKD is a significant contributor to hospital mortality in AE-IPF, and diabetes mellitus may be protective. Obesity and hypothyroidism are linked with shorter hospital LOS among patients hospitalized with AE-IPF in US academic medical centers.

## 1. Introduction

Idiopathic pulmonary fibrosis (IPF) is a chronic progressive lung disease of unknown cause with a high associated mortality. It is often characterized as a disease of the aging lung, most often presenting in men above the age of 60 [1,2]. In this older population, increasing consideration has been given to the role of multimorbidity in the progression and prognosis of chronic disease states such as IPF [3,4]. Although comorbidities such as pulmonary hypertension, chronic obstructive pulmonary disease, lung cancer, gastroesophageal reflux, and ischemic heart disease have been identified as prevalent among patients with IPF, their impact on health outcomes and mortality is less well-defined [4].

Even less is known about the role of comorbidity specifically in acute exacerbations of IPF (AE-IPF). AE-IPF, most recently reframed as an “acute, clinically significant respiratory deterioration characterized by evidence of new widespread alveolar abnormality”, contributes to as many as 40% of IPF deaths [5,6]. High body mass index, pulmonary hypertension, and cardiovascular disease are chronic medical conditions that have been explored as risk factors for the development of AE-IPF [7,8,9]. Laboratory, physiologic, and radiographic parameters at the time of AE-IPF have been used to predict survival, but the contribution of chronic comorbidity in this acute setting is unknown [10,11,12]. Therefore, using a national database, we aimed to identify chronic comorbidities that may be associated with poor outcomes among hospitalized patients with AE-IPF. We hypothesized that certain common chronic comorbidities significantly contribute to poorer outcomes in patients hospitalized with AE-IPF.

## 2. Materials and Methods 

### 2.1. Study Design and Data Source 

This was a retrospective study analyzing patient discharge information from the Healthcare Cost and Utilization Project’s (HCUP) National Inpatient Sample (NIS), an initiative of the Agency for Healthcare Research and Quality (AHRQ) [13]. This extensive database, accessible to the public in the United States, captures over seven million annual hospital stays from 48 states, representing 97% of the US population since 2012. The NIS data, which are weighted to accurately reflect nationwide discharge trends, include a broad sample from all hospitals. Each record in the NIS provides detailed information, including up to 40 diagnosis codes and 15 procedure codes per stay, based on the *International Classification of Diseases, 10th Revision, Clinical Modification* (ICD-10-CM). The first out of forty assigned codes is the principal diagnosis and the rest are secondary codes thereafter in numerical order. In addition to anonymized patient data, some hospital attributes are also available. Due to the de-identified and publicly accessible nature of the HCUP data, we did not request IRB approval or patient consent.

### 2.2. Inclusion and Exclusion Criteria

Our cohort included adult patients aged 60 and older hospitalized with AE-IPF between January 2016 and December 2018. Previous studies have used claims data to identify IPF cases but have employed ICD-9-CM diagnostic coding [14,15]. Since our cohort is a contemporary cohort, we isolated cases using the ICD 10 code J84.112 (specific diagnosis code for IPF) combined with a diagnosis code for acute or acute on chronic respiratory failure (ICD-10 CM codes J96.00, J96.01, J96.02, J96.20, J96.21, J96.22, J96.90, J96.91, and J96.92). We included all patients with the first two (principal and first secondary) codes of either IPF or acute respiratory failure. As the gold standard for IPF diagnosis involves a multidisciplinary approach most likely to be fully realized at specialty centers, we limited our analyses to patients admitted to academic medical centers [16]. This approach ensured the capture of patients who were likely to have AE-IPF with new or worsening respiratory failure. Elective admissions were excluded from analysis as we aimed to focus on acute respiratory failure necessitating non-elective medical care. We excluded interhospital transfers as we could not reliably calculate total hospital length of stay (LOS). 

### 2.3. Patient and Variables

We collected patient and hospital characteristics (Table 1 and Table 2) from the NIS, aiming to investigate risk factors linked to the primary outcome, in-hospital mortality. We explored hospital LOS as a secondary outcome. The predictors of interest were age, gender, race, income status, insurance status, hospital bed size, hospital location, year of admission, number of ICD-10-CM diagnoses in the medical record, number of billed procedures during hospitalization, and common chronic medical conditions administratively identified using ICD-10-CM codes (Supplementary Table S1), including hypertension, diabetes mellitus, obesity, hypothyroidism, alcohol and drug use, chronic kidney disease (CKD), cardiovascular disease, and malignancy.

### 2.4. Statistical Analysis 

In assessing patient characteristics, we applied different statistical methods: Student’s *t*-test for data with normal distribution, Mann–Whitney U test for data not normally distributed, and Pearson chi-square test for categorical data. The NIS dataset guidelines led us to adopt survey-weighted analyses. To determine the odds ratios (aOR), we used multiple logistic regression, controlling for variables such as age, gender, race, household income, payer status, hospital bed size, location, admission year, number of diagnoses, procedures, and relevant chronic medical conditions. We experimented with various modeling approaches, including forward stepwise conditional, backward stepwise conditional, and the enter method. As there was no significant variance in outcomes related to AE-IPF mortality, we have chosen to present the results from the enter method. Additionally, we conducted negative binomial regression to pinpoint risk factors for increased hospital length of stay (LOS), adjusting for the same factors. Statistical significance was set at a two-sided *p* value of less than 0.05. IBM SPSS Statistics for Windows, version 25 (IBM Corp., Armonk, NY, USA), was used for all analyses.

## 3. Results 

**Figure 1:**
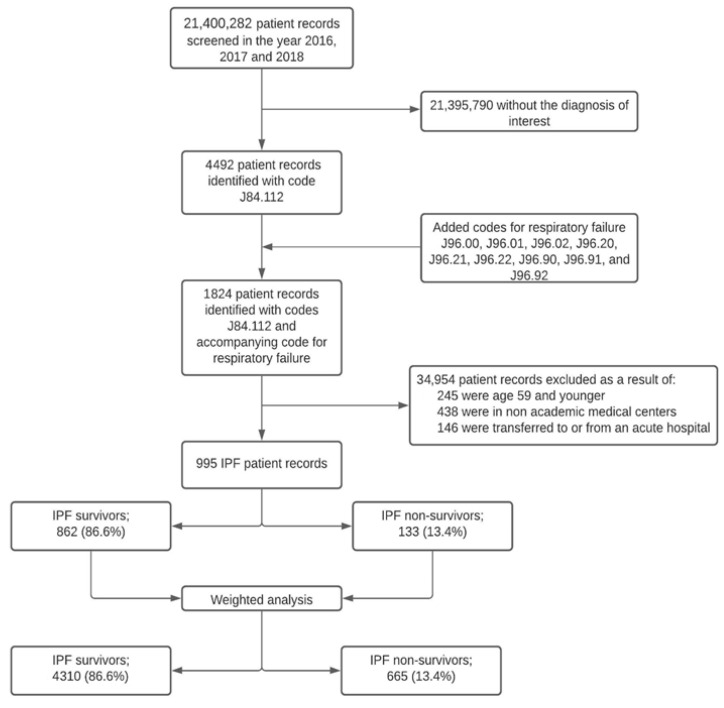
Cohort selection.

Out of the 1824 patients identified with IPF exacerbation using the ICD-10-CM diagnosis code of J84.112 and accompanying code for respiratory failure, 829 did not meet the inclusion criteria (See Figure 1 for cohort selection). The remaining 995 cases were split into two cohorts for analysis: survivors (862 cases) and non-survivors (133 cases). When considering survey-weighted data, the count of cases in these respective groups was 4310 and 665, respectively. 

The characteristics of the patients and hospitals, divided based on mortality outcomes, are shown in Table 1. The average age was comparable between the two groups (74.0 vs. 74.5 years; *p* = 0.14). A higher proportion of patients were male (57.8% vs. 69.9%; *p* < 0.001), and the majority were white, with a statistically significant difference in racial composition (*p* < 0.001). Notable differences were observed in the average number of diagnoses (16.4 vs. 18.5; *p* < 0.001) and procedures (2.0 vs. 2.5; *p* = 0.001) between the groups. Medicare was the predominant coverage in both cohorts. Higher median household incomes were more common among non-survivors.

**Figure 2:**
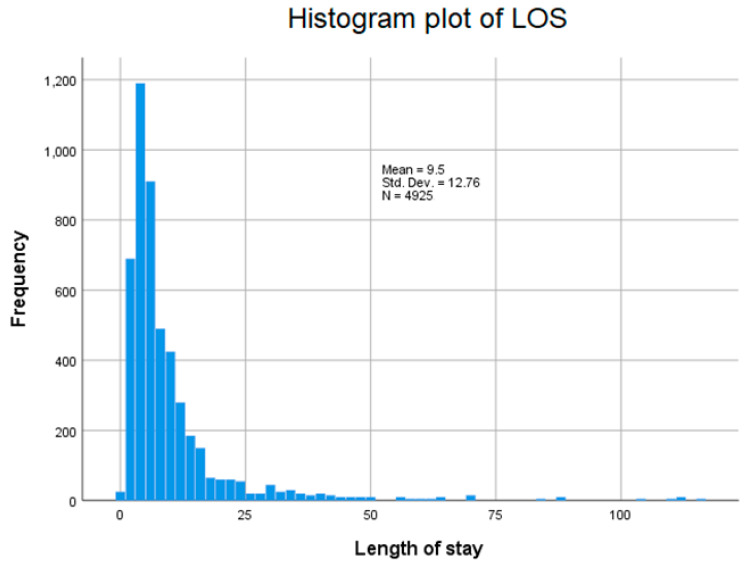
Histogram plot of length of stay.

The distribution of chronic medical conditions is shown in Table 1. Among the predictors examined, there were differences in the distribution of diabetes, hypothyroidism, and CKD between the two groups. The distribution of the hospital LOS for all patients is shown in Figure 2 (mean hospital LOS = 9.5 days). Hospital LOS was observed to be longer among non-survivors compared to survivors (9.4 days vs. 9.8 days; *p* < 0.001).

Hospital characteristics stratified by survivor status are displayed in Table 2. The distribution of patients across the three years was not uniform, with 2018 showing the largest percentage of patients (39.3% vs. 39.1%) among survivors and non-survivors. Larger academic facilities with more beds admitted more than 50% of the patients. About 35% of patients received care in the Southern region (Table 2).

Results of the logistic regression model examining chronic medical comorbidities as risk factors for death in hospitalized patients with AE-IPF are presented in Table 3. All covariates used in risk adjustment are shown in Supplementary Table S2. We found that the presence of diabetes was protective (aOR 0.62, 95% CI 0.50–0.77; *p* < 0.0001), while CKD conferred a significantly higher risk of death after AE-IPF (aOR 6.85, 95% CI 1.90–24.7; *p* = 0.00). For every 10-year increase in age, the risk of mortality increased (aOR 1.24, 95% CI 1.09–1.40; *p* < 0.0001). The female sex was associated with lower odds of in-hospital death compared to the male sex (aOR 0.67, 95% CI 0.55–0.82; *p* < 0.0001; Supplementary Table S2).

For the outcome of hospital LOS and its association with chronic comorbidities, the results of negative binomial regression are presented in Table 4. All covariates used in the model are again shown in Supplementary Table S2. Obesity (IRR 0.85, 95% CI 0.76–0.94; *p* ≤ 0.00) and hypothyroidism (IRR 0.90, 95% CI 0.83–0.98; *p* = 0.02) were associated with a shorter hospital LOS. For every 10-year increase in age, the risk of longer hospital LOS decreased (IRR 0.91, 95% CI 0.87–0.95; *p* < 0.0001).

## 4. Discussion

### 4.1. Summary of Results

Our results show that among common chronic medical comorbidities, CKD confers a significantly higher risk of hospital death after AE-IPF. In contrast, comorbid diabetes mellitus is associated with lower odds of death. Among other covariates included in fully adjusted models, older age is associated with a higher risk of death and an accordingly shorter LOS during hospitalization for AE-IPF. For the secondary outcome of hospital LOS, metabolic conditions such as obesity and hypothyroidism were associated with shorter durations of hospitalization.

### 4.2. Comparison with Previously Published Data

Multimorbidity as measured by the Charlson Comorbidity Index (CCI) has previously been examined for its potential role as a prognostic factor in the progression of stable interstitial lung disease [17]. A composite prognostic score including the CCI has also been studied in acute and subacute idiopathic interstitial pneumonia and acute exacerbations of collagen vascular disease-related interstitial pneumonia [18]. In the latter cohort, the authors found that there was no difference between survivors and non-survivors of AE-IPF in the summed score of the 19 comorbidities encompassed by the CCI [18,19]. However, our population-based study identifies specific comorbidities (namely, CKD and diabetes mellitus) that impact AE-IPF prognosis.

The association of CKD with increased risk of mortality in hospitalized IPF patients has previously been noted [20]. The mechanism for the increased risk of death after AE-IPF associated with CKD is unknown. Ikezoe et al. reported that a high proportion of patients with IPF have comorbid CKD, and a lower estimated glomerular filtration rate is associated with worse survival in IPF [21]. A recent autopsy study of 12 patients called attention to the fact that AE-IPF has systemic consequences culminating in multiple organ injury, including the kidneys [22]. It is possible that the strikingly high prevalence of CKD in IPF predisposes or perhaps even potentiates the likelihood of the pathologic necrosis of renal tubular epithelial cells seen on autopsy by Emura and Usada, thereby contributing to an increased risk of death during hospitalization for AE-IPF [21,22].

It is interesting to note the seeming protective effect of diabetes on survival in this population. The role of diabetes in AE-IPF prognosis is not well studied and may be more related to the degree of glycemic control rather than the diagnosis alone. For instance, fasting glucose and glycosylated hemoglobin have shown a significant negative association with lung function parameters such as forced vital capacity, diffusion of carbon monoxide of the lung, and total lung capacity among patients with interstitial lung disease [23]. That said, the pathophysiology of AE-IPF has been likened to that of acute respiratory distress syndrome (ARDS) [24]. Several studies have shown the incidence of ARDS to be lower among diabetics, and it is possible that diabetes may similarly reduce the risk of AE-IPF [25,26]. The prevalence of pulmonary fibrosis has also been noted to be lower in diabetic decedents compared to non-diabetic decedents in a large population-based study [27]. Yet the previous literature does not suggest a relationship between diabetes and ARDS mortality [28,29]. A potential mechanism for our findings is that the overall lower incidence of diabetes in the IPF population reduces the risk of associated diabetic pneumopathy, thereby contributing to a lower risk of death in AE-IPF [30]. Further investigation into the mechanism by which diabetes or the degree of diabetic control may protect against death during hospitalization for AE-IPF is necessary in order to explain this phenomenon.

The underlying factors contributing to the observed association between hypothyroidism and reduced LOS during hospitalization for AE-IPF remain uncertain. To our knowledge, there are no data exploring the impact of hypothyroidism specifically on AE-IPF outcomes. Large-scale genomic investigations have revealed that hypothyroidism may exert a positive causal influence on IPF [31]. Hypothyroidism has been noted to be both highly prevalent and a predictor of reduced survival in IPF patients overall [32]. It is possible that the association of shorter hospital LOS with the hypothyroid cohort may be attributable to increased mortality.

Similarly, the association between obesity and reduced LOS during AE-IPF hospitalization may also be related, in part, to mortality, albeit with opposite directionality. Awano et al. found higher body mass index (BMI) to be associated with better survival during AE-IPF [33]. An “obesity paradox” has been proposed in IPF, whereby obese patients with IPF exhibit improved mortality outcomes [34]. Weight loss has been associated with worse survival in IPF [35]. Addressing obesity-related traits (e.g., BMI) could even potentially serve as a preventative measure against IPF development [36]. It is plausible that the reduced hospital LOS observed in obese patients with AE-IPF in our study may be indicative of a generally milder disease severity in this population and better survival.

### 4.3. Strengths and Limitations

Our study addresses a gap in knowledge by identifying comorbidities associated with poor prognosis specifically in hospitalized patients with AE-IPF. The burden of comorbidities is noted to be high among IPF patients, and increasing number of comorbidities has been associated with worse survival [37]. However, to our knowledge, our study is unique in examining the impact of comorbidity on mortality in AE-IPF. Moreover, the use of more contemporary data enables the analysis of inpatient outcomes in AE_IPF during the era of antifibrotic therapy. Despite the slower-than-anticipated adoption of antifibrotic treatments, existing evidence suggests a connection between the use of these treatments and enhanced survival rates [38,39]. Our research also newly identifies a previously unreported protective effect of diabetes on hospital mortality related to AE-IPF, which is a novel finding.

There are several limitations to our study. Firstly, we used ICD-10-CM codes to select patients. It is possible that our use of the ICD 10 code J84.112 affected case selection such that potentially relevant cases were either not fully or overly captured. There is limited data on the sensitivity and specificity of ICD-based codes for IPF, and available studies have shown that administrative codes perform poorly [40]. Jeganathan et al. were able to show the impact of diagnostic coding selection on the generation of contradictory IPF mortality trend results using the same dataset as Dove and colleagues [41,42]. Moreover, there has been observed a heterogeneity of outcome in acute exacerbations of idiopathic interstitial pneumonias overall, and this, combined with the absence of an administrative case definition for IPF, may have impacted our results [43]. However, using our selection criteria, we observed similar a hospital mortality rate (13.4%) among patients with AE-IPF as that noted by Durheim et al. [20]. It is also possible that fewer patients are being labelled with IPF due to the evolution of diagnostic criteria or reclassification [44]. In their review of 445 patients with IPF using the revised definition, Yoo et al. found an in-hospital mortality following AE-IPF of 29.5%, which is higher than what we found but much lower than previously reported [45]. Global variability in the treatment of AE-IPF may also contribute to our US population-based findings and limit the generalizability of our findings [46]. Lastly, survival after AE-IPF may simply have improved over time, as has survival for ARDS with better adherence to low-tidal volume ventilatory strategies and general advances in critical care [47]. Antifibrotic treatment, which reduces the risk of AE-IPF, may also have an impact on AE-IPF-related mortality in a more contemporary dataset [48].

## 5. Conclusions

Recent reviews have recognized the importance of managing comorbidities, both pulmonary and extra-pulmonary, among patients with IPF [49,50]. Whereas the inflammatory milieu of accelerated aging may foster the development or potentiation of comorbidities, there is a paucity of data examining the impact of individual comorbidities on outcomes after hospitalization for AE-IPF [51]. Our results add to the existing literature by extending the implications of chronic comorbidities beyond overall IPF mortality and grounding them in a specific clinical scenario, namely AE-IPF.

Our results suggest that CKD is a significant contributor to hospital mortality in AE-IPF, and diabetes mellitus may be protective. The inclusion of comorbidities has been shown to improve the prediction of all-cause mortality in IPF [52]. We anticipate that our results may also contribute to our limited understanding of contributors to prognosis at the time of hospitalization for AE-IPF. With a more granular examination of the directionality and mechanism of impact of discrete comorbidities on IPF outcomes, researchers may begin to modify disease progression prior to hospitalization for AE-IPF or perhaps even prevent the development of AE-IPF, thereby impacting survival. Therefore, further study of the role of multimorbidity specifically in outcomes after AE-IPF may have therapeutic value in both prevention and management.

## Figures and Tables

**Table 1:** Patient characteristics.

Variable	IPF—Survivors	IPF—Non-Survivors	*p*-Value
N	4310 (86.6%)	665 (13.4%)	
**Age in years, Mean (SD)**	74.0 (8.0)	74.5 (7.6)	0.14
**Sex—male N (%)**	2490 (57.8%)	465 (69.9%)	<0.001
**Race, N (%)**			<0.001
White	3080 (73.8%)	495 (78.0%)	
Black	295 (7.1%)	40 (6.3%)	
Hispanic	550 (13.2%)	45 (7.1%)	
Other	250 (6.0%)	55 (8.7%)	
**Median household income, N (%)**			<0.001
Very low	855 (20.1%)	125 (19.2%)	
Low	1030 (24.2%)	120 (18.5%)	
Medium	1225 (28.8%)	135 (20.8%)	
High	1145 (26.9%)	270 (41.5%)	
**Primary payer, N (%)**			<0.0001
Medicare	3530 (81.9%)	485 (72.9%)	
Medicaid	145 (3.4%)	25 (3.8%)	
Private/HMO	525 (12.2%)	130 (19.5%)	
Other/self-pay	110 (2.6%)	25 (3.8%)	
**Number of diagnoses, Mean (SD)**	16.4 (6.1)	18.5 (5.3)	<0.001
Number of procedures, Mean (SD) *	2.0 (4.1)	2.5 (3.5)	<0.001
**Chronic medical conditions**			
Hypertension	2860 (66.4%)	440 (66.2%)	0.92
Diabetes	1280 (29.7%)	150 (22.6%)	<0.001
Obesity	480 (11.1%)	60 (9.0%)	0.10
Hypothyroidism	840 (19.5%)	105 (15.8%)	0.02
Alcohol and drug use	125 (2.9%)	15 (2.9%)	0.35
Chronic kidney disease	5 (0.1%)	5 (0.8%)	0.001
Cardiovascular disease	385 (8.9%)	50 (7.5%)	0.23
Malignancy	185 (4.3%)	25 (3.8%)	0.53
**Discharge disposition**			
Home	1715 (39.8%)		
Skilled nursing facility	1185 (27.5%)		
Home with home healthcare	1410 (32.7%)		
**Length of stay in days, Mean (SD)** *	9.4 (13.2)	9.8 (9.1)	<0.001

All analyses done based on survey-weighted N. * Non-parametric test (Mann–Whitney U) was used.

**Table 2:** Hospital characteristics.

Variable	IPF—Survivors	IPF—Non-Survivors	*p*-Value
**Admission year, N (%)**			0.02
2016	1260 (29.2%)	225 (33.8%)	
2017	1355 (31.4%)	180 (27.1%)	
2018	1695 (39.3%)	260 (39.1%)	
**Bed size of hospital, N (%)**			0.54
Large	2350 (54.5%)	375 (56.4%)	
Medium	1155 (26.8%)	165 (24.8%)	
Small	805 (18.7%)	125 (18.8%)	
**Region of hospital, N (%)**			0.00
Northeast	1000 (23.2%)	195 (29.3%)	
Midwest	875 (20.3%)	110 (16.5%)	
South	1565 (36.3%)	230 (34.6%)	
West	870 (20.2%)	130 (19.5%)	

All analyses done based on survey-weighted N.

**Table 3:** Multivariate logistic regression of predictors of in-hospital IPF mortality.

Chronic Medical Conditions	aOR	95% CI	*p*-Value
Hypertension	1.03	0.84–1.25	0.80
Diabetes	0.62	0.50–0.77	<0.001
Obesity	0.82	0.60–1.12	0.22
Hypothyroidism	0.81	0.63–1.04	0.10
Alcohol and drug use	0.58	0.33–1.05	0.07
Chronic kidney disease	6.85	1.90–24.70	0.00
Cardiovascular disease	0.74	0.54–1.01	0.06
Malignancy	0.74	0.48–1.17	0.20

Analysis is adjusted for age, gender, race, primary payer, median household income, number of diagnoses and procedures, hospital region, bed size, and calendar year of admission.

**Table 4:** Multivariate negative binomial regression of predictors of hospital LOS in exacerbated IPF patients.

Chronic Medical Conditions	Incidence Rate Ratio (IRR)	95% CI	*p*-Value
Hypertension	0.93	0.87–1.00	0.06
Diabetes	1.02	0.95–1.10	0.55
Obesity	0.85	0.76–0.94	0.00
Hypothyroidism	0.90	0.83–0.98	0.02
Alcohol and drug use	1.11	0.92–1.33	0.28
Chronic kidney disease	1.14	0.59–2.21	0.70
Cardiovascular disease	0.96	0.86–1.07	0.50
Malignancy	1.09	0.93–1.27	0.28

Analysis is adjusted for age, gender, race, primary payer, median household income, number of diagnoses and procedures, hospital region, bed size, and calendar year of admission.

## Data Availability

The National Inpatient Sample Database is available through Agency for Healthcare Research and Quality.

## Supplementary Materials

The following are available online at https://www.mdpi.com/article/10.3390/life14010156/s1, Table S1: Diagnostic codes used to identify chronic medical conditions, Table S2: Adjusted odds ratio (aOR) and Incidence rate ratios (IRR) of the factors used to adjust for analyzing impact of chronic comorbidities on in-hospital mortality and hospital LOS.

## Abbreviations

Idiopathic pulmonary fibrosis (IPF). Acute exacerbation of idiopathic pulmonary fibrosis (AE-IPF). National Inpatient Sample (NIS). Length of stay (LOS). Chronic kidney disease (CKD). United States of America (US).
