# Supplementary material for: The Impact of Chronic Comorbidities on Outcomes in Acute Exacerbations of Idiopathic Pulmonary Fibrosis
Source: Life (Basel). 2024 Jan 21;14(1):156. doi: 10.3390/life14010156 (PMC10817308; doi:10.3390/life14010156)
Supplement: Supplementary file 1 [file life-14-00156-s001.zip › life-2813784-supplementary.pdf]

life

MDPI

Study Protocol

The Impact of Chronic Comorbidities on Outcomes in Acute Exacerbations of Idiopathic Pulmonary Fibrosis

Saqib H. Baig [1,*] and Erika J. Yoo [1]

[1] Division of Pulmonary, Allergy and Critical Care Medicine, Jane and Leonard Korman Respiratory Institute, Sidney Kimmel Medical College, Thomas Jefferson University, 211 South 9th Street, Suite 401, Philadelphia, PA 19107, USA; erika.yoo@jefferson.edu

* Correspondence: saqib.baig@jefferson.edu; Tel.: +1-443-825-9106

Table S1. Diagnostic codes used to identify chronic medical conditions.

**Hypertension**	**H35031, H35032, H35033, H35039, I110, I119, I120, I129, I130, I1310, I1311, I132, I150, I151, I152, I158, I159, I161, I674, O10111, O10112, O10113, O10119, O1012, O1013, O10211, O10212, O10213, O10219, O1022, O1023, O10311, O10312, O10313, O10319, O1032, O1033, O10411, O10412, O10413, O10419, O1042, O1043, O10911, O10912, O10913, O10919, O1092, O1093, O111, O112, O113, O114, O115, O119, O161, O162, O163, O164, O165, O169, I10, I160, I169, O10011, O10012, O10013, O10019, O1002, O1003**
Diabetes	E08XXX – E13XXX<br>O24011, O24012, O24013, O24019, O2402, O2403, O24111, O24112, O24113, O24119, O2412, O2413, O24311, O24312, O24313, O24319, O2432, O2433, O24410, O24414, O24415, O24419, O24420, O24424, O24425, O24429, O24430, O24434, O24435, O24439, O24811, O24812, O24813, O24819, O2482, O2483, O24911, O24912, O24913, O24919, O2492, O2493
Obesity	E6601, E6609, E661, E662, E668, E669, O99210, O99211, O99212, O99213, O99214, O99215, R939, Z6830, Z6831, Z6832, Z6833, Z6834, Z6835, Z6836, Z6837, Z6838, Z6839, Z6841, Z6842, Z6843, Z6844, Z6845, Z6854
Hypothyroidism	E000, E001, E002, E009, E010, E011, E012, E018, E02, E030, E031, E032, E033, E034, E035, E038, E039, E890
Alcohol and drug abuse	F10XXX – F19XXX<br>G621, I426, K2920, K2921, K7010, K7011, O99310, O99311, O99312, O99313, O99314, O99315, O99320, O99321, O99322, O99323, O99324, O99325, O99320, O99321, O99322, O99323, O99324, O99325
Chronic kidney disease	I120, I1311, I132, N184, N185, N186, Z4901, Z4902, Z4931, Z4932, Z9115, Z940, Z992, N183,<br>N1830, N1831, N1832, N189, N19
Cardiovascular disease	I20XX – I25XX<br>I60XX – I69XX<br>I70XX – I75XX<br>P91821, P91822, P91823, P91829, G450, G451, G452, G453, G454, G458, G459, G460, G461, G462, G463, G464, G465, G466, G467, G468, H3400, H3401, H3402, H3403, H3410, H3411, H3412, H3413, H34211, H34212, H34213, H34219, H34231, H34232, H34233, H34239
Malignancy	C00XXX – C80XXX<br>C7AXX<br>D469, E3121, E3122, E3123

Table S2. Adjusted odds ratio (aOR) and Incidence rate ratios (IRR) of the factors used to adjust for analyzing impact of chronic comorbidities on in-hospital mortality and hospital LOS.

	In-hospital mortality			Hospital LOS		
**Variable (Details on comparison group)**	**aOR**	**95% CI**	**p-value**	**IRR**	**95% CI**	**p-value**
Age (for 10-year increase)	1.24	1.09–1.40	0.00	0.91	0.87–0.95	< 0.001
Gender (Male)	0.67	0.55–0.82	< 0.001	1.02	0.96–1.10	0.48
Race (White)						
Black	0.91	0.63–1.31	0.61	1.02	0.90–1.16	0.76
Asian	0.59	0.42–0.84	0.00	1.05	0.94–1.16	0.35
Other	1.29	0.93–1.80	0.13	1.06	0.93–1.20	0.41
Income status (Very low-income group)						
Low-income group	0.72	0.54–0.96	0.02	0.98	0.89–1.08	0.74
Moderate-income group	0.60	0.45–0.80	< 0.001	1.08	0.98–1.18	0.11
High-income group	1.19	0.92–1.54	0.18	1.08	0.98–1.19	0.13
Insurance status (Medicare)						
Medicaid	2.54	1.56–4.15	< 0.001	0.99	0.83–1.99	0.94
Private	1.90	1.48–2.45	< 0.001	1.10	1.00–1.21	0.06
Other/self pay	1.98	1.22–3.20	0.00	0.90	0.74–1.10	0.30
Calendar year (2016)						
Year 2017	0.72	0.58–0.91	0.00	0.93	0.86–1.01	0.09
Year 2018	0.83	0.68–1.03	0.08	0.86	0.80–0.93	< 0.001
Number of procedures	0.98	0.96–1.01	0.19	1.10	1.09–1.11	< 0.001
Number of diagnosis codes	1.08	1.06–1.10	< 0.001	1.04	1.03–1.05	< 0.001
Academic hospital bed size (small)						
Medium bed size	0.90	0.69–1.18	0.44	1.04	0.94–1.14	0.46
Large bed size	0.88	0.69–1.12	0.30	1.15	1.05–1.25	0.00
Hospital region (Northwest)						
Midwest	0.80	0.61–1.06	0.12	0.71	0.65–0.78	< 0.001
South	0.96	0.76–1.21	0.73	0.84	0.77–0.91	< 0.001
West	0.86	0.66–1.13	0.28	0.71	0.65–0.78	< 0.001
